# Association of Race and Ethnicity With Obstructive Coronary Artery Disease

**DOI:** 10.1016/j.jacadv.2022.100161

**Published:** 2023-01-11

**Authors:** Jasjit Rooprai, Feng Qiu, Joan Porter, Husam Abdel-Qadir, Lucas C. Godoy, Cynthia A. Jackevicius, Douglas S. Lee, Mina Madan, Baiju R. Shah, Maneesh Sud, Harindra C. Wijeysundera, Dennis T. Ko

**Affiliations:** a ICES, Toronto, Ontario, Canada; b Institute of Health Policy, Management and Evaluation, University of Toronto, Ontario, Canada; c Department of Medicine, University of Toronto, Ontario, Canada; d Peter Munk Cardiac Centre of University Health Network, Toronto, Ontario, Canada; e Ted Rogers Centre for Heart Research, Toronto, Ontario, Canada; f Women's College Hospital, Toronto, Ontario, Canada; g Instituto do Coracao (InCor), Hospital das Clinicas HCFMUSP, Faculdade de Medicina, Universidade de Sao Paulo, São Paulo, Brazil; h College of Pharmacy, Western University of Health Sciences, Pomona, California, USA; i Veterans Affairs Greater Los Angeles Healthcare System, Los Angeles, California, USA; j Schulich Heart Centre, Sunnybrook Health Sciences Centre, Toronto, Ontario, Canada; k Department of Medicine, Sunnybrook Health Sciences Centre, Toronto, Ontario, Canada

**Keywords:** coronary angiography, coronary artery disease, ethnicity, race

## Abstract

**Background:**

Appropriate selection of patients with stable coronary artery disease (CAD) for coronary angiography is dependent on the pretest probability of obstructive CAD; however, little is known about the potential differences in CAD by race and ethnic groups.

**Objectives:**

The purpose of this study was to evaluate the association of race and ethnicity with coronary obstruction in stable CAD.

**Methods:**

We evaluated first coronary angiography for CAD evaluation between 2012 and 2019 in Ontario, Canada. Race and ethnicity were identified by physicians. The main outcome was the rate of obstructive CAD (left main stenosis ≥50% or major epicardial vessel stenosis ≥70%). Multivariable logistic regression analyses evaluated the independent association of race and ethnicity with CAD.

**Results:**

Among 71,199 CAD patients, 14.0% were South Asian (SA), 4.4% were East Asian (EA), and 58,131 were White patients. SA patients were the youngest at 60.9 years vs 62.4 years for EA patients and 65.1 years for White patients but were most likely to have obstructive CAD (46.9%) (EA 43.0% and White patients 37.9%). SA patients had the highest prevalence of 3-vessel CAD at 13.4% (vs 12.5% in EA and 7.7% in White patients). The adjusted odds ratio was 67% higher (1.67; 95% CI: 1.59 to 1.75) for having obstructive CAD in SA patients than that in White patients. EA patients also had significantly increased adjusted odds of obstructive CAD compared with White patients (1.40; 95% CI: 1.29-1.52).

**Conclusions:**

SA patients were younger at presentation but had the highest adjusted odds of obstructive CAD. Incorporation of race and ethnicity information may improve risk-prediction tools for detection of coronary obstruction.

An increasing number of individuals of South Asian (India, Pakistan, Bangladesh, Sri Lanka, and Nepal) and East Asian (China, Japan, Vietnam, Korea, Hong Kong, and Taiwan) race and ethnicity reside in North America.^1^, ^2^, ^3^, ^4^, ^5^ In 2019, it was estimated that 5.6 million South Asian residents and 15.5 million East Asian residents reside in the United States.^1^ It is further estimated that the Asian population will more than double to ∼46 million in the United States by 2060.^2^ Even a higher proportion of South Asian and East Asian individuals reside in Canada. It is estimated that 1.9 million South Asian residents and 2.95 million East Asian residents reside in Canada, where they represent 5.5% and 8.6% of the population, respectively.^3^ Studies evaluating South Asians' health have found a higher prevalence of cardiac risk factors, including hyperlipidemia and diabetes, than that in the general population.^5^, ^6^, ^7^, ^8^, ^9^ They have also been found to be at higher risk of developing acute myocardial infarction and dying from cardiovascular causes.^5^, ^6^, ^7^, ^8^, ^9^ In contrast, studies that evaluated East Asian patients have shown a lower prevalence of cardiovascular risk factors and a lower incidence of acute myocardial infarction than that in the general population.^10^^,^^11^ However, there remain many gaps of knowledge. One of the main limitations of data from the United States if the fact that South Asian and East Asian individuals are frequently considered as a single group despite their vastly different cardiovascular risk profiles.^12^, ^13^, ^14^ Other limitations in the literature included assembling cohorts only with private health insurance coverage, which could introduce selection bias.

It is estimated that 18.2 million Americans and 2.5 million Canadians are currently living with stable coronary artery disease (CAD).^15^ Selecting appropriate patients for coronary angiography is dependent on understanding the pretest probability of obstructive CAD. The original Diamond and Forrester model predicts obstructive CAD based on age, sex, and symptoms.^16^ Newer predictive risk scores include cardiac risk factors such as diabetes, hypertension, dyslipidemia, and smoking status and have found improved discrimination ability.^17^ Yet, no study to our knowledge thus far have fully evaluated the potential influence of race and ethnicity in detecting obstructive coronary stenosis in a stable CAD cohort. Accordingly, the main objective of our study was to evaluate the association of race and ethnicity with the detection of obstructive CAD by comparing the incidence of obstructive CAD among South Asians, East Asians, and White patients undergoing initial diagnostic coronary angiography.

## Methods

### Data sources

We conducted a retrospective cohort study using population-linked administrative, clinical, and laboratory data in the province of Ontario, Canada. The CorHealth Ontario Cardiac Registry is a prospective clinical database that collects data on demographic, clinical, and procedural characteristics of all patients undergoing cardiac procedures in the province. Invasive cardiac care is regionalized in Ontario to 18 centers serving a provincial population of more than 14 million individuals. This clinical registry was linked to: 1) the Canadian Institute for Health Information Discharge Abstract Database for additional clinical comorbidities; 2) the Ontario Laboratories Information System for laboratory results; 3) the Ontario Drug Benefit program for information about prescription drug use for patients older than 65 years; and 4) census data to ascertain socioeconomic status. Additional details on these databases can be found in prior work.^18^^,^^19^

### Study population

Our study cohort included adult patients undergoing their first coronary angiography for a diagnosis of stable CAD between April 1, 2012, and March 31, 2019. The diagnosis of CAD was based on the clinicians' assessment and noninvasive ischemia testing as coronary computed tomography angiographies are performed infrequently in Ontario. We excluded patients with a prior cardiovascular disease (history of myocardial infarction or unstable angina, cerebrovascular disease, peripheral vascular disease) and those with prior coronary revascularization (a percutaneous coronary intervention or a coronary artery bypass graft surgery). For patients who had multiple cardiac catheterizations during the study period, only the first procedure was considered.

### Identification of South Asian and East Asian patients

Race and ethnicity information was obtained by the CorHealth registry, which captured data recorded by referring physicians at the time of coronary angiography referral. The CorHealth definition of South Asian patients was individuals of Indian, Pakistani, Sri Lankan, or Bangladeshi descent or culture, while East Asian patients were defined as those of Chinese, Japanese, Vietnamese, Korean, or Taiwanese descent or culture.

### Outcome variables

The primary outcome of the study was the presence of obstructive CAD, defined as a stenosis of ≥50% in the left main coronary artery or stenosis of ≥70% in a major epicardial coronary artery (left anterior descending coronary artery, left circumflex coronary artery, and right coronary artery).^18^^,^^19^ Fractional flow reserve results were not required in the diagnosis of obstructive CAD. We also examined diseases in individual artery segments, as well as the number of vessels with obstructive disease, using definitions consistent with prior research. Coronary anatomy from this database has been previously validated.^18^^,^^19^

### Statistical analyses

We compared demographic characteristics, clinical characteristics, and the extent of obstructive CAD in 3 different groups (South Asian, East Asian, and White patients). Analysis of variance was used for the comparison of continuous variables, and chi-square analysis was used to compare categorical variables.

Multivariable logistic regression modeling was used to estimate the independent association of race and ethnicity with the incidence of coronary obstructive CAD. Variables included in the model were demographics (age, sex) and conventional cardiac risk factors (diabetes, hypertension, current smoking, former smoking, and high-density lipoprotein [HDL] cholesterol and non-HDL cholesterol levels within the past 3 years). These factors were chosen as they are known to be associated with coronary obstruction and cardiovascular events.^20^ We performed several additional analyses to evaluate the association of race and ethnicity with obstructive CAD in several predefined subgroups: age (<60, ≥60 years), sex, income quintile, and angina severity as defined by the Canadian Cardiovascular Society classification.

Data sets were linked using unique encoded identifiers and analyzed at ICES (formerly known as the Institute for Clinical Evaluative Sciences). SAS version 9.2 (SAS Institute Inc) was used for statistical analyses. A 2-sided *P* value of 0.05 or less was considered statistically significant in the comparison of outcomes. The use of data in this project is authorized under section 45 of Ontario’s Personal Health Information Protection Act and does not require review by a research ethics board.

## Results

### Study sample

**Figure 1:**
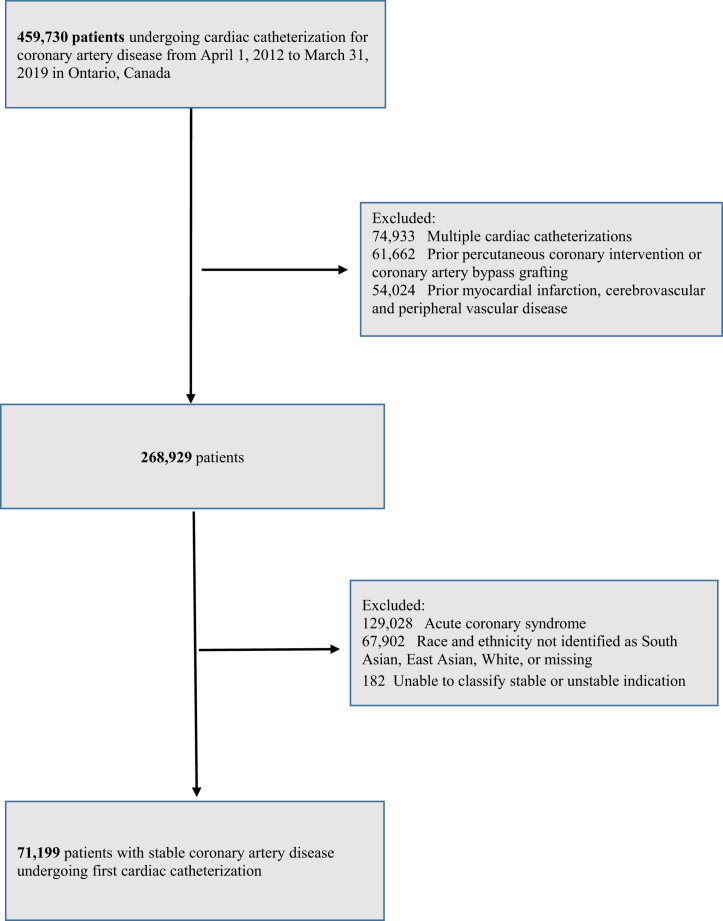
**Construction of the Study Cohort** A total of 459,730 cardiac catheterizations were performed for the indication of coronary artery disease over the period April 1, 2012, to March 31, 2019. After inclusion and exclusion criteria were applied, our final study cohort consisted of 71,199 adult patients

The construction of the study cohort is shown in Figure 1. There were 459,730 cardiac catheterizations performed for the indication of CAD over the period April 1, 2012, to March 31, 2019. We excluded 61,662 patients because of prior coronary revascularization, 54,024 patients for prior cardiovascular conditions, 74,933 repeat coronary angiograms in the study period, and 129,028 patients because the procedure was performed for an acute coronary syndrome. After excluding those without race and ethnicity data, our final study cohort consisted of 71,199 adult patients of which 9,938 (14.0%) were categorized as South Asian, 3,130 (4.4%) as East Asian, and 58,131 (81.6%) as White patients.

### Baseline characteristics

Table 1 shows the baseline characteristics of the study cohort. South Asian patients were one and a half years younger (mean age 60.9 years) than East Asian patients (62.4 years) and 4 years younger than White patients (65.1 years). South Asian patients had the highest prevalence of diabetes (50.9% vs 38.4% in East Asian and 30.3% in White patients), hypertension (68.9% vs 67.0% East Asian and 66.8% White patients), and hyperlipidemia (68.8% vs 59.9% East Asian and 56.7% White patients). In contrast, South Asian patients were less likely than the other ethnic groups to smoke (7.1% vs 9.0% East Asians vs 14.0% Whites). They were also more likely to have angina (74.5%) with Canadian Cardiovascular Society Class 1 or more symptoms than East Asian (63.6%) and White (60.3%) patients. Supplemental Tables 1 and 2 show baseline characteristics by gender, race, and ethnicity. The overall trend was similar in the sex-specific analysis. For example, age of presentation to cardiac catheterization in men and women was youngest among South Asians patients.

**Table 1:** Baseline Characteristics of Patients Undergoing Cardiac Catheterization by Race and Ethnic Group

	South Asian (n = 9,938)	East Asian (n = 3,130)	White (n = 58,131)	*P* Value
Demographics				
Age, y	60.9 ± 11.20	62.4 ± 11.80	65.1 ± 11.73	<0.001
Male	6,212 (62.5)	1,984 (63.4)	34,826 (59.9)	<0.001
Rural residency	31 (0.3)	23 (0.7)	7,994 (13.8)	<0.001
Socioeconomic status				
Lowest neighborhood income quintile	2,056 (20.7)	712 (22.7)	10,728 (18.5)	<0.001
Highest neighborhood income quintile	988 (9.9)	524 (16.7)	12,621 (21.7)	<0.001
Cardiac risk factors and comorbidities				
Diabetes	5,054 (50.9)	1,201 (38.4)	17,600 (30.3)	<0.001
Hypertension	6,849 (68.9)	2,097 (67.0)	38,813 (66.8)	<0.001
Dyslipidemia	6,838 (68.8)	1,876 (59.9)	32,972 (56.7)	<0.001
Current smoker	701 (7.1)	281 (9.0)	8,151 (14.0)	<0.001
Former smoker	1,071 (10.8)	560 (17.9)	18,360 (31.6)	<0.001
Heart failure	412 (4.1)	293 (9.4)	5,352 (9.2)	<0.001
Chronic obstructive pulmonary disease	125 (1.3)	56 (1.8)	3,579 (6.2)	<0.001
Canadian Cardiovascular Society angina classification				
Class 0	2,535 (25.5)	1,139 (36.4)	23,058 (39.7)	<0.001
Class I	2,088 (21.0)	511 (16.3)	9,344 (16.1)	<0.001
Class II	3,788 (38.1)	977 (31.2)	16,475 (28.3)	<0.001
Class III	999 (10.1)	316 (10.1)	5,274 (9.1)	0.002
Class IV	66 (0.7)	21 (0.7)	458 (0.8)	0.351
Body mass index, kg/m^2^				
<18.5	88 (0.9)	47 (1.5)	404 (0.7)	<0.001
18.5–<25	2,256 (22.7)	980 (31.3)	8,162 (14.0)	<0.001
25–<30	3,071 (30.9)	820 (26.2)	12,620 (21.7)	<0.001
30–<35	1,304 (13.1)	217 (6.9)	8,470 (14.6)	<0.001
35–<40	353 (3.6)	57 (1.8)	3,616 (6.2)	<0.001
≥40	174 (1.8)	29 (0.9)	2,528 (4.3)	<0.001
Serum creatinine, mg/dL				
<1.36	8,941 (90.0)	2,742 (87.6)	52,027 (89.5)	<0.001
1.36-2.04	438 (4.4)	175 (5.6)	3,313 (5.7)	<0.001
>2.04	242 (2.4)	107 (3.4)	1,055 (1.8)	<0.001
Left ventricular ejection fraction				
<20%	89 (0.9)	68 (2.2)	1,035 (1.8)	<0.001
20%-34%	260 (2.6)	149 (4.8)	3,293 (5.7)	<0.001
35%-49%	571 (5.7)	249 (8.0)	5,629 (9.7)	<0.001
≥50%	7,926 (79.8)	2,318 (74.1)	39,496 (67.9)	<0.001
Graded exercise stress test				
Test performed	6,090 (61.3)	1,667 (53.3)	26,891 (46.3)	<0.001
High-risk results	2,585 (26.0)	749 (23.9)	10,101 (17.4)	<0.001
Low-risk results	2,920 (29.4)	767 (24.5)	13,639 (23.5)	<0.001
Functional imaging ischemia test				
Test performed	6,147 (61.9)	1,650 (52.7)	27,304 (47.0)	<0.001
High-risk results	2,853 (28.7)	738 (23.6)	11,560 (19.9)	<0.001
Low-risk results	3,079 (31.0)	847 (27.1)	14,602 (25.1)	<0.001
Cholesterol value in the past 3 y, mmol/L^a^				
Total cholesterol	4.35 ± 1.16	4.44 ± 1.17	4.55 ± 1.21	<0.001
Non-HDL-cholesterol	3.14 ± 1.12	3.14 ± 1.13	3.23 ± 1.14	<0.001
LDL-cholesterol	2.39 ± 0.97	2.40 ± 0.98	2.51 ± 1.01	<0.001
HDL-cholesterol	1.21 ± 0.34	1.30 ± 0.38	1.31 ± 0.41	<0.001
Triglycerides	1.71 ± 1.15	1.70 ± 1.11	1.66 ± 1.20	<0.001
Blood glucose (HbA1c) in the past 3 y mmol/L^b^	6.69 ± 1.34	6.45 ± 1.26	6.16 ± 1.14	<0.001
Fasting glucose in the past 3 y mmol/L^c^	6.53 ± 2.22	6.27 ± 2.18	6.26 ± 2.09	<0.001
Medication use for patients aged >65 y in the past 90 d prior to procedure^d^				
Angiotensin converting enzyme inhibitor	1,006 (28.0)	286 (22.4)	9,511 (32.5)	<0.001
Angiotensin receptor blocker	1,338 (37.3)	472 (37.0)	7,077 (24.2)	<0.001
Beta-blocker	1,818 (50.6)	569 (44.6)	13,356 (45.7)	<0.001
Statin	2,576 (71.8)	826 (64.7)	16,985 (58.1)	<0.001
Oral diabetic medication	1,409 (39.2)	365 (28.6)	5,848 (20.0)	<0.001
Insulin	331 (9.2)	89 (7.0)	1,608 (5.5)	<0.001

Values are mean ± SD or n (%).

HDL = high-density lipoprotein; LDL = low-density lipoprotein.

a Cholesterol information available on 63,874 patients (89.7% of cohort).

b HbA1C information available on 58,314 patients (81.9% of cohort).

c Fasting glucose information available on 47,892 (67.3% of cohort).

d Medication information based on 3,590 South Asians patients, 1,276 East Asian patients, and 29,232 White patients older than 65 years.

Cholesterol values were available for 90% of the study cohort within 3 years of cardiac catheterization. South Asian and East Asian patients had similar low-density lipoprotein cholesterol levels (2.39 ± 0.97 mmol/L vs 2.40 ± 0.98 mmol/L), which were slightly lower than 2.51 ± 1.01 mmol/L in Whites. Among patients with diabetes, South Asians had the highest HbA1c (6.69 ± 1.34 mmol/L), followed by East Asian (6.45 ± 1.26 mmol/L) and White patients (6.16 ± 1.14 mmol/L). For patients older than 65 years (where medication information was available), South Asian patients were most likely to be on angiotensin receptor blockers, beta blockers, statins, and antihyperglycemic medications (oral antihyperglycemic agents and insulin).

### Presence of a CAD

As shown in Table 2, South Asian patients had the highest prevalence of any obstructive CAD (46.9%) compared with East Asian (43.0%) and White (37.9%) patients. Obstructive left main disease was found to be more prevalent among East Asian patients (5.4% East Asians vs 4.1% South Asians vs 4.0% Whites), whereas the three-vessel disease was more prevalent among South Asian patients (13.4% South Asians vs 12.5% East Asians vs 7.7% Whites).

**Table 2:** Obstructive Coronary Artery Disease for Patients Undergoing First Cardiac Catheterization by Race and Ethnic Group^a^

	South Asian (n = 9,938)	East Asian (n = 3,130)	White (n = 58,131)	*P* Value
Presence of obstructive coronary artery disease				
Obstructive coronary artery disease	4,665 (46.9)	1,347 (43.0)	22,059 (37.9)	<0.001
Coronary artery disease location				
Left main artery ≥50%	406 (4.1)	168 (5.4)	2,333 (4.0)	<0.001
Proximal left anterior descending artery ≥70%	1,534 (15.4)	504 (16.1)	6,715 (11.6)	<0.001
Mid to distal left anterior descending artery ≥70%	2,947 (29.7)	819 (26.2)	11,590 (19.9)	<0.001
Circumflex artery ≥70%	2,582 (26.0)	718 (22.9)	10,210 (17.6)	<0.001
Right coronary artery ≥70%	2,568 (25.8)	709 (22.7)	11,803 (20.3)	<0.001
Number of diseased major vessel excluding those with left main stenosis				
0 vessel disease	5,301 (53.3)	1,799 (57.5)	36,371 (62.6)	<0.001
1 vessel disease	1,881 (18.9)	579 (18.5)	10,929 (18.8)	<0.001
2 vessel disease	1,429 (14.4)	361 (11.5)	6,354 (10.9)	<0.001
3 vessel disease	1,327 (13.4)	391 (12.5)	4,477 (7.7)	<0.001

Values are n (%).

a Obstructive coronary artery disease was defined as a stenosis of ≥50% in the left main coronary artery or stenosis of ≥70% in a major epicardial coronary artery (left anterior descending coronary artery, left circumflex coronary artery, and right coronary artery).

### Association of race and ethnicity with obstructive CAD

The association of race and ethnicity with obstructive CAD adjusting for demographic and cardiac risk factors is summarized in Table 3. Patients of South Asian decent had an adjusted odds ratio (aOR) of 1.67 (95% CI: 1.59-1.75) for obstructive CAD compared with White patients. East Asian ethnicity was associated with a 40% higher risk of obstructive CAD (aOR 1.40, 95% CI: 1.29-1.52) than White patients. Traditional cardiac risk factors, such as diabetes (aOR 1.52, 95% CI: 1.46-1.58), current smoking status (aOR 1.46, 95% CI: 1.38-1.54), and non-HDL cholesterol levels (aOR 1.33, 95% CI: 1.31-1.35) were also found to be independently associated with obstructive CAD. In contrast, higher HDL-cholesterol levels were associated with lower odds of CAD (aOR 0.63, 95% CI: 0.60-0.67).

**Table 3:** Multivariable Model of the Association of Race and Ethnicity and Obstructive Coronary Artery Disease

	Odds Ratio (95% CI)	*P* Value
Race/ethnicity		
South Asian vs White (as reference)	1.67 (1.59-1.75)	<0.0001
East Asian vs White (as reference)	1.40 (1.29-1.52)	<0.0001
Demographics		
Age (per y)	1.05 (1.04-1.05)	<0.0001
Female (male as reference)	0.355 (0.342-0.369)	<0.0001
Cardiac risk factors		
Diabetes	1.52 (1.46-1.58)	<0.0001
Hypertension	1.16 (1.11-1.20)	<0.0001
Current smoker	1.46 (1.38-1.54)	<0.0001
Former smoker	1.12 (1.08-1.17)	<0.0001
HDL-cholesterol levels (per mmol/L)	0.63 (0.60-0.67)	<0.0001
Non HDL-cholesterol levels (per mmol/L)	1.33 (1.31-1.35)	<0.0001

Odds ratio adjusted for age, sex, and conventional cardiac risk factors (diabetes, hypertension, current smoking, former smoking, HDL-cholesterol levels, and non-HDL cholesterol within the past 3 years).

HDL = high-density lipoprotein.

### Prevalence of obstructive cad and aOR in prespecified subgroups

Table 4 shows rates of obstructive CAD and the aOR of the association of race and ethnic groups with obstructive CAD. In prespecified subgroups by age, sex, income, and angina categories, similar patterns were observed for higher incidence of CAD among South Asian and East Asian patients than among White patients.

**Table 4:** Crude Proportion and Adjusted Odds Ratio of Obstructive CAD in Major Epicardial Vessels by Race and Ethnicity and Subgroup

	Rate of CAD			Adjusted Odds Ratio (95% CI)	
	South Asian (n = 9,938)	East Asian (n = 3,130)	White (n = 58,131)	South Asian vs White	East Asian vs White
Age group, y					
<60	1,616/4,388 (36.8)	453/1,247 (36.3)	5,147/18,275 (28.2)	1.46 (1.35-1.58)	1.63 (1.43-1.86)
≥60	3,049/5,550 (54.9)	894/1,883 (47.5)	16,912/39,856 (42.4)	1.62 (1.52-1.72)	1.28 (1.16-1.41)
Sex					
Male	3,507/6,212 (56.5)	1,045/1,984 (52.7)	16,153/34,826 (46.4)	1.39 (1.31-1.47)	1.27 (1.16-1.40)
Female	1,158/3,726 (31.1)	302/1,146 (26.4)	5,906/23,305 (25.3)	1.27 (1.17-1.38)	1.14 (0.99-1.32)
Neighborhood income quintile					
Lower quintile (1-3)	3,435/7,337 (46.8)	865/2,043 (42.3)	9,296/34,008 (38.5)	1.42 (1.34-1.50)	1.28 (1.17-1.41)
Medium to high (quintile 4-5)	1,230/2,601 (47.3)	482/1,087 (44.3)	12,763/24,123 (37.5)	1.34 (1.23-1.47)	1.33 (1.16-1.51)
CCS angina classification					
0-1	1,962/4,623 (42.4)	567/1,650 (34.4)	10,479/32,402 (32.3)	1.43 (1.34-1.53)	1.12 (1.00-1.25)
2 or above	2,691/5,279 (51.0)	773/1,456 (53.1)	11,396/25,296 (45.1)	1.25 (1.17-1.33)	1.46 (1.31-1.64)

Values are n/N (%) unless otherwise indicated.

Odds ratio adjusted for age, sex, and conventional cardiac risk factors (diabetes, hypertension, current smoking, former smoking, high-density lipoprotein [HDL] cholesterol levels, and non-HDL cholesterol within the past 3 years).

CAD = coronary artery disease; CCS = Canadian Cardiovascular Society.

## Discussion

In this cohort of patients with stable CAD undergoing coronary angiography for their initial diagnosis, we found substantial differences in demographic and clinical characteristics between race and ethnicity groups. Although South Asian patients were the youngest group at presentation, prevalence of obstructive CAD and severe multivessel CAD was significantly higher than that in other race and ethnicity groups. Even after adjusting for age, sex, and traditional cardiac risk factors, race and ethnicity were independently associated with the presence of obstructive CAD, where South Asians had a 67% higher aOR and East Asians had 40% higher odds of having obstructive CAD than White patients. The magnitude of the association of obstructive CAD with race and ethnicity was similar to that of the association with traditional risk factors, such as diabetes or smoking. Our findings suggest that it is important to consider race and ethnicity information in selecting patients for cardiac catheterization.

Many studies have focused primarily on the race and ethnicity difference in the development of acute cardiac conditions such as acute myocardial infarction.^5^^,^^11^^,^^21^^,^^22^ For example, in a cohort of 824,662 immigrants to Canada, Tu et al^11^ found South Asian immigrants had the highest rates of acute myocardial infarction compared with other race and ethnic groups. These differences were attributed to a higher rate of diabetes, less favorable cholesterol profiles (higher triglycerides, higher low-density lipoprotein cholesterol, lower HDL-cholesterol), and also increased inflammatory and prothrombotic markers in South Asians.^11^^,^^23^, ^24^, ^25^ We were able to extend these findings by examining a cohort of stable CAD cases undergoing cardiac catheterization and adjusted for cardiac risk factors and cholesterol levels and found persistent difference in the rate of obstructive CAD. We also observed a higher use of cardiac and antihyperglycemic medications among older South Asian patients to manage these risk factors. However, we were unable to evaluate whether South Asian and East Asian patients received optimal care in primary prevention given the lack of information on factors including diet, physical activity, and social determinants of health.

In addition to these factors, other reasons that could contribute to the discordant rates of obstructive CAD among race and ethnic groups include health behaviors such as dietary habits and exercise, socioeconomic status, and the acculturation impact on immigrants. We were unable to account for health-seeking behaviors, and it is possible preventive treatment for primary cardiac prevention may have differed between groups.

More than 4 decades ago, Diamond and Forrester^16^ found prediction of CAD was possible based on the patients’ age, sex, and symptoms of chest pain. Their model is widely recommended and has been adopted into clinical practice guidelines.^24^^,^^25^ Newer models have been developed, but none has considered race and ethnicity of the patients. Given the independent association of race and ethnicity with CAD, our finding suggests that future models that incorporate race and ethnicity could improve the discrimination (ie, the ability of a model to differentiate between patients with and without disease) in predicting obstructive CAD. Concordant with our findings, the latest guidelines on chest pain suggest potential differences in presentation based on patient’s race and ethnicity and recommend cultural competency training to help achieve the best outcomes in the evaluation of patients with chest pain.^26^

### Study Limitations

Our findings should be interpreted in the context of several potential limitations. First, even though we had access to data on all coronary angiography procedures in the province of Ontario, it is possible that more obstructive CAD cases in 1 race and ethnic group was due to referral for evaluation later in the course of disease. While we could not exclude this possibility, we observed South Asian patients had the highest rate of obstructive CAD but also had the youngest age of presentation. Second, information on race and ethnicity in our study was based on data obtained by physicians during the referral for cardiac catheterization, and thus, it was not standardized. It has been advocated that race-ethnicity determination based on patient self-identification is optimal because it would allow for a more accurate determination and allows a patient to identify multiple race and ethnicity categories. Third, our study focused on the prevalence of obstructive CAD because we believe that would allow for an objective primary endpoint in our study in evaluating patients with stable CAD. Nevertheless, our finding may not be generalizable to those with stable angina not having an invasive evaluation. Fourth, we were unable to evaluate potential differences within the South Asian and East Asian patients because our data did not capture country of origin of each patient.

## Conclusions

Our findings show substantial race and ethnic differences in cardiac risk factors and presence of obstructive CAD (Central Illustration). In addition, we found that race and ethnicity are independently associated with the presence of obstructive CAD and are more influential than traditional risk factors. This highlights the importance of race and ethnicity in considering the pretest probability of CAD and in selecting patients with stable ischemic heart disease for referral for cardiac catheterization.

PERSPECTIVES **COMPETENCY IN PATIENT CARE:** In this large study of stable patients undergoing coronary angiography, we found that South Asian patients were younger at presentation but had the highest adjusted odds of obstructive disease.

**TRANSLATIONAL OUTLOOK:** Incorporation of race and ethnicity information may improve risk-prediction tools for detection of coronary obstruction.

**Central Illustration:**
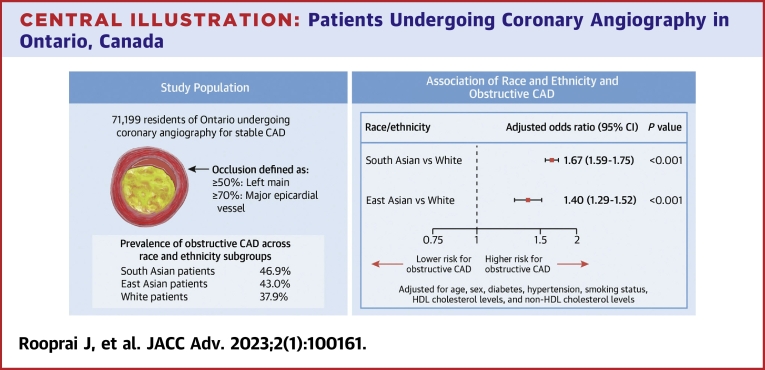
**Patients Undergoing Coronary Angiography in Ontario, Canada** The main outcome was the rate of obstructive coronary artery disease (CAD) (left main stenosis ≥50% or major epicardial vessel stenosis ≥70%). The prevalence of obstructive CAD was highest in South Asian patients at 46.9%, followed by East Asian patients at 43.0%, and White patients at 37.9%. The adjusted odds ratio of having obstructive CAD was 67% higher than that for White patients. East Asian patients also had significant 40% adjusted odds of obstructive CAD compared with White patients. HDL = high-density lipoprotein.

## Funding support and author disclosures

This study was supported by 10.13039/100012665ICES, which is funded by an annual grant from the 10.13039/501100000226Ontario Ministry of Health and the Ministry of Long-Term Care. This study is funded in part by Foundation grants (FDN 154333) by the 10.13039/501100000024Canadian Institutes of Health Research (CIHR). The authors acknowledge that the clinical registry data used in this publication are from participating hospitals through CorHealth Ontario, which serves as an advisory body to the Minister of Health and Long-Term Care (MOHLTC), is funded by the MOHLTC, and is dedicated to improving the quality, efficiency, access, and equity in the delivery of the continuum of adult cardiac, vascular, and stroke services in Ontario, Canada. Dr Godoy has received the Frederick Banting and Charles Best Canada Graduate Scholarship (Doctoral Research Award) from the Canadian Institutes of Health Research. Dr Ko is supported by the Jack Tu Research Chair in Cardiovascular Outcomes Research, Sunnybrook hospital, and University of Toronto. All other authors have reported that they have no relationships relevant to the contents of this article to disclose.

## References

[1] (2021). Key facts about Asian Americans, a diverse and growing population. https://www.pewresearch.org/fact-tank/2021/04/29/key-facts-about-asian-americans/.

[2] (2019). 2019 American Community Survey 1-year estimates. https://www.census.gov/quickfacts/fact/table/US/PST045219.

[3] Visible minority population, by province and territory (2006 Census). https://www12.statcan.gc.ca/census-recensement/2006/rt-td/eth-eng.cfm.

[4] (2017). Key facts about Asian Americans, a diverse and growing population. https://www.pewresearch.org/fact-tank/2017/09/08/key-facts-about-asian-americans/.

[5] Anand S.S., Yusuf S., Vuksan V. (2000). Differences in risk factors, atherosclerosis, and cardiovascular disease between ethnic groups in Canada: the Study of Health Assessment and Risk in Ethnic groups (SHARE). Lancet.

[6] Fischbacher C.M., Bhopal R., Povey C. (2007). Record linked retrospective cohort study of 4.6 million people exploring ethnic variations in disease: myocardial infarction in South Asians. BMC Public Health.

[7] Gupta M., Singh N., Verma S. (2006). South Asians and cardiovascular risk: what clinicians should know. Circulation.

[8] Reddy K.S., Prabhakaran D., Jeemon P. (2007). Educational status and cardiovascular risk profile in Indians. Proc Natl Acad Sci U S A.

[9] Reddy K.S., Yusuf S. (1998). Emerging epidemic of cardiovascular disease in developing countries. Circulation.

[10] Chiu J.F., Bell A.D., Herman R.J. (2010). Cardiovascular risk profiles and outcomes of Chinese living inside and outside China. Eur J Cardiovasc Prev Rehabil.

[11] Tu J.V., Chu A., Rezai M.R. (2015). The incidence of major cardiovascular events in immigrants to Ontario, Canada: the CANHEART immigrant study. Circulation.

[12] Mital R., Bayne J., Rodriguez F., Ovbiagele B., Bhatt D.L., Albert M.A. (2021). Race and ethnicity Considerations in patients with coronary artery disease and stroke: JACC Focus Seminar 3/9. J Am Coll Cardiol.

[13] Pursnani S., Merchant M. (2020). South Asian ethnicity as a risk factor for coronary heart disease. Atherosclerosis.

[14] Yusuf S., Reddy S., Ounpuu S., Anand S. (2001). Global burden of cardiovascular diseases: part II: variations in cardiovascular disease by specific ethnic groups and geographic regions and prevention strategies. Circulation.

[15] Virani S.S., Alonso A., Aparicio H.J. (2021). Heart disease and stroke statistics-2021 update: a report from the American Heart Association. Circulation.

[16] Diamond G.A., Forrester J.S. (1979). Analysis of probability as an aid in the clinical diagnosis of coronary-artery disease. N Engl J Med.

[17] Genders T.S., Steyerberg E.W., Hunink M.G. (2012). Prediction model to estimate presence of coronary artery disease: retrospective pooled analysis of existing cohorts. BMJ.

[18] Tu J.V., Ko D.T., Guo H. (2012). Determinants of variations in coronary revascularization practices. CMAJ.

[19] Sud M.H.L., Koh M., Austin P.C. (2020). Association between adherence to fractional flow reserve treatment thresholds and major adverse cardiac events in patients with coronary artery disease. JAMA.

[20] Wilson P.W., D'Agostino R.B., Levy D., Belanger A.M., Silbershatz H., Kannel W.B. (1998). Prediction of coronary heart disease using risk factor categories. Circulation.

[21] Chi G.C., Kanter M.H., Li B.H. (2020). Trends in acute myocardial infarction by race and ethnicity. J Am Heart Assoc.

[22] Graham G. (2016). Racial and ethnic differences in acute coronary syndrome and myocardial infarction within the United States: from demographics to outcomes. Clin Cardiol.

[23] Anand S.S., Razak F., Yi Q. (2004). C-reactive protein as a screening test for cardiovascular risk in a multiethnic population. Arterioscler Thromb Vasc Biol.

[24] Fihn S.D., Gardin J.M., Abrams J. (2012). 2012 ACCF/AHA/ACP/AATS/PCNA/SCAI/STS guideline for the diagnosis and management of patients with stable ischemic heart disease: a report of the American College of Cardiology Foundation/American Heart Association task force on practice guidelines, and the American College of Physicians, American Association for Thoracic Surgery, Preventive Cardiovascular Nurses Association, Society for Cardiovascular Angiography and Interventions, and Society of Thoracic Surgeons. J Am Coll Cardiol..

[25] Task Force M., Montalescot G., Sechtem U. (2013). 2013 ESC guidelines on the management of stable coronary artery disease: the task force on the management of stable coronary artery disease of the European Society of Cardiology. Eur Heart J.

[26] Writing Committee M., Gulati M., Levy P.D. (2021). 2021 AHA/ACC/ASE/CHEST/SAEM/SCCT/SCMR guideline for the evaluation and diagnosis of chest pain: a report of the American College of Cardiology/American Heart Association Joint Committee on clinical practice guidelines. J Am Coll Cardiol.

