# Supplementary material for: Association of Race and Ethnicity With Obstructive Coronary Artery Disease
Source: JACC Adv. 2023 Jan 11;2(1):100161. doi: 10.1016/j.jacadv.2022.100161 (PMC11198462; doi:10.1016/j.jacadv.2022.100161)
Supplement: Supplemental Tables 1 and 2 [file mmc1.docx]

| **Supplemental Table 1. Baseline characteristics of female patients undergoing cardiac catheterization by race and ethnic group** | | | | |
| --- | --- | --- | --- | --- |
|  | **South Asian** | **East Asian** | **White** | **p-value** |
|  | **N=3,726** | **N=1,146** | **N=23,305** |  |
| **Demographics, n (%)** |  |  |  |  |
| Mean age in years ± SD | 62.13 ± 11.15 | 63.93 ± 11.81 | 66.40 ± 11.73 | <.001 |
| Rural residency | 9 (0.2) | 11 (1.0) | 3,109 (13.3) | <.001 |
| **Socioeconomic status, n (%)** |  |  |  |  |
| Lowest neighborhood income quintile | 809 (21.7) | 291 (25.4) | 4,839 (20.8) | <.001 |
| Highest neighborhood income quintile | 334 (9.0) | 164 (14.3) | 4,432 (19.0) | <.001 |
| **Cardiac risk factors and comorbidities, n (%)** | |  |  |  |
| Diabetes | 1,895 (50.9) | 439 (38.3) | 7,048 (30.2) | <.001 |
| Hypertension | 2,672 (71.7) | 786 (68.6) | 16,209 (69.6) | 0.019 |
| Dyslipidemia | 2,474 (66.4) | 646 (56.4) | 12,837 (55.1) | <.001 |
| Current smoker | 54 (1.4) | 30 (2.6) | 2,740 (11.8) | <.001 |
| Former smoker | 63 (1.7) | 42 (3.7) | 5,741 (24.6) | <.001 |
| Heart failure | 177 (4.8) | 130 (11.3) | 2,088 (9.0) | <.001 |
| Chronic obstructive pulmonary disease | 44 (1.2) | 15 (1.3) | 1,555 (6.7) | <.001 |
| **Canadian Cardiovascular Society angina classification, n (%)** | |  |  |  |
| Class 0 | 871 (23.4) | 425 (37.1) | 8,982 (38.5) | <.001 |
| Class I | 732 (19.6) | 179 (15.6) | 3,649 (15.7) | <.001 |
| Class II | 1,489 (40.0) | 351 (30.6) | 6,796 (29.2) | <.001 |
| Class III | 412 (11.1) | 122 (10.6) | 2,328 (10.0) | 0.115 |
| Class IV | 35 (0.9) | 7 (0.6) | 196 (0.8) | 0.562 |
| **Body mass index (kg/m^2^), n (%)** |  |  |  |  |
| < 18.5 | 44 (1.2) | 22 (1.9) | 261 (1.1) | 0.047 |
| 18.5 to < 25 | 742 (19.9) | 393 (34.3) | 3,665 (15.7) | <.001 |
| 25 to < 30 | 1,049 (28.2) | 240 (20.9) | 4,225 (18.1) | <.001 |
| 30 to < 35 | 628 (16.9) | 78 (6.8) | 3,251 (13.9) | <.001 |
| 35 to < 40 | 208 (5.6) | 30 (2.6) | 1,745 (7.5) | <.001 |
| ≥ 40 | 98 (2.6) | 14 (1.2) | 1,314 (5.6) | <.001 |
| **Serum creatinine (mg/dL), n (%)** |  |  |  |  |
| < 1.36 mg/dL | 3,477 (93.3) | 1,041 (90.8) | 21,462 (92.1) | 0.007 |
| 1.36 to 2.04 mg/dL | 88 (2.4) | 40 (3.5) | 832 (3.6) | <.001 |
| > 2.04 mg/dL | 55 (1.5) | 31 (2.7) | 292 (1.3) | <.001 |
| **Left Ventricular Ejection Fraction, n (%)** |  |  |  |  |
| < 20% | 23 (0.6) | 19 (1.7) | 276 (1.2) | 0.002 |
| 20 to 34% | 92 (2.5) | 57 (5.0) | 1,006 (4.3) | <.001 |
| 35 to 49 % | 164 (4.4) | 59 (5.1) | 1,694 (7.3) | <.001 |
| ≥50% | 3,048 (81.8) | 884 (77.1) | 17,035 (73.1) | <.001 |
| **Graded exercise stress test, n (%)** |  |  |  |  |
| Test performed | 2,044 (54.9) | 537 (46.9) | 10,063 (43.2) | <.001 |
| High risk results | 817 (21.9) | 204 (17.8) | 3,510 (15.1) | <.001 |
| Low risk results | 984 (26.4) | 281 (24.5) | 5,224 (22.4) | <.001 |
| **Functional imaging ischemia test, n (%)** |  |  |  |  |
| Test performed | 2,341 (62.8) | 580 (50.6) | 11,214 (48.1) | <.001 |
| High risk results | 1,055 (28.3) | 220 (19.2) | 4,464 (19.2) | <.001 |
| Low risk results | 1,205 (32.3) | 338 (29.5) | 6,302 (27.0) | <.001 |
| **Mean cholesterol value in the past 3 years, mmol/L ± SD*** |  |  |  |  |
| Total cholesterol | 4.47 ± 1.11 | 4.58 ± 1.12 | 4.78 ± 1.21 | <.001 |
| Non-HDL cholesterol | 3.14 ± 1.09 | 3.15 ± 1.08 | 3.31 ± 1.15 | <.001 |
| LDL-cholesterol | 2.42 ± 0.96 | 2.41 ± 0.96 | 2.61 ± 1.04 | <.001 |
| HDL-cholesterol | 1.33 ± 0.35 | 1.44 ± 0.40 | 1.47 ± 0.43 | <.001 |
| Triglycerides | 1.62 ± 0.92 | 1.69 ± 1.06 | 1.61 ± 1.00 | 0.022 |
|  |  |  |  |  |
| **Mean blood glucose (HbA1c) in the past 3 years mmol/L ± SD**** | 6.71 ± 1.28 | 6.47 ± 1.29 | 6.14 ± 1.10 | <.001 |
| **Mean fasting glucose in the past 3 years mmol/L ± SD ***** | 6.49 ± 2.23 | 6.19 ± 2.17 | 6.14 ± 2.06 | <.001 |
|  |  |  |  |  |
| **Medication use for patients > 65 years in the past 90 days prior to procedure, n (%) ****** |  |  |  |  |
| Angiotensin converting enzyme inhibitor | 388 (25.7) | 106 (20.1) | 3,713 (29.0) | <.001 |
| Angiotensin receptor blocker | 626 (41.5) | 203 (38.5) | 3,469 (27.1) | <.001 |
| Beta Blocker | 781 (51.8) | 230 (43.6) | 5,893 (46.0) | <.001 |
| Statin | 1,069 (70.9) | 334 (63.4) | 7,066 (55.2) | <.001 |
| Oral diabetic medication | 607 (40.3) | 141 (26.8) | 2,335 (18.2) | <.001 |
| Insulin | 154 (10.2) | 44 (8.3) | 648 (5.1) | <.001 |
| Abbreviation: SD, standard deviation |  |  |  |  |
| * Total cholesterol information available on 25,272 patients (89.7% of female cohort) | | | |  |
| ** HbA1C information available on 23,236 patients (82.5% of cohort) | |  |  |  |
| *** Fasting glucose information available on 19,054 (67.6% of female cohort) | |  |  |  |
| **** Medication information based on 1,508 South Asians patients, 527 East Asian patients, and 12,805 White patients older than 65 years | | | |  |

| **Supplemental Table 2. Baseline characteristics of male patients undergoing cardiac catheterization by race and ethnic group** | | | | |
| --- | --- | --- | --- | --- |
|  | **South Asian** | **East Asian** | **White** | **p-value** |
|  | **N=6,212** | **N=1,984** | **N=34,826** |  |
| **Demographics, n (%)** |  |  |  |  |
| Mean age in years ± SD | 60.19 ± 11.17 | 61.58 ± 11.71 | 64.14 ± 11.65 | <.001 |
| Rural residency | 22 (0.4) | 12 (0.6) | 4,885 (14.0) | <.001 |
| **Socioeconomic status, n (%)** |  |  |  |  |
| Lowest neighborhood income quintile | 1,247 (20.1) | 421 (21.2) | 5,889 (16.9) | <.001 |
| Highest neighborhood income quintile | 654 (10.5) | 360 (18.1) | 8,189 (23.5) | <.001 |
| **Cardiac risk factors and comorbidities, n (%)** | |  |  |  |
| Diabetes | 3,159 (50.9) | 762 (38.4) | 10,552 (30.3) | <.001 |
| Hypertension | 4,177 (67.2) | 1,311 (66.1) | 22,604 (64.9) | 0.001 |
| Dyslipidemia | 4,364 (70.3) | 1,230 (62.0) | 20,135 (57.8) | <.001 |
| Current smoker | 647 (10.4) | 251 (12.7) | 5,411 (15.5) | <.001 |
| Former smoker | 1,008 (16.2) | 518 (26.1) | 12,619 (36.2) | <.001 |
| Heart failure | 235 (3.8) | 163 (8.2) | 3,264 (9.4) | <.001 |
| Chronic obstructive pulmonary disease | 81 (1.3) | 41 (2.1) | 2,024 (5.8) | <.001 |
| **Canadian Cardiovascular Society angina classification, n (%)** | |  |  |  |
| Class 0 | 1,664 (26.8) | 714 (36.0) | 14,076 (40.4) | <.001 |
| Class I | 1,356 (21.8) | 332 (16.7) | 5,695 (16.4) | <.001 |
| Class II | 2,299 (37.0) | 626 (31.6) | 9,679 (27.8) | <.001 |
| Class III | 587 (9.4) | 194 (9.8) | 2,946 (8.5) | 0.007 |
| Class IV | 31 (0.5) | 14 (0.7) | 262 (0.8) | 0.092 |
| **Body mass index (kg/m^2^), n (%)** |  |  |  |  |
| < 18.5 | 44 (0.7) | 25 (1.3) | 143 (0.4) | <.001 |
| 18.5 to < 25 | 1,514 (24.4) | 587 (29.6) | 4,497 (12.9) | <.001 |
| 25 to < 30 | 2,022 (32.5) | 580 (29.2) | 8,395 (24.1) | <.001 |
| 30 to < 35 | 676 (10.9) | 139 (7.0) | 5,219 (15.0) | <.001 |
| 35 to < 40 | 145 (2.3) | 27 (1.4) | 1,871 (5.4) | <.001 |
| ≥ 40 | 76 (1.2) | 15 (0.8) | 1,214 (3.5) | <.001 |
| **Serum creatinine (mg/dL), n (%)** |  |  |  |  |
| < 1.36 mg/dL | 5,464 (88.0) | 1,701 (85.7) | 30,565 (87.8) | 0.022 |
| 1.36 to 2.04 mg/dL | 350 (5.6) | 135 (6.8) | 2,481 (7.1) | <.001 |
| > 2.04 mg/dL | 187 (3.0) | 76 (3.8) | 763 (2.2) | <.001 |
| **Left Ventricular Ejection Fraction, n (%)** |  |  |  |  |
| < 20% | 66 (1.1) | 49 (2.5) | 759 (2.2) | <.001 |
| 20 to 34% | 168 (2.7) | 92 (4.6) | 2,287 (6.6) | <.001 |
| 35 to 49 % | 407 (6.6) | 190 (9.6) | 3,935 (11.3) | <.001 |
| ≥50% | 4,878 (78.5) | 1,434 (72.3) | 22,461 (64.5) | <.001 |
| **Graded exercise stress test, n (%)** |  |  |  |  |
| Test performed | 4,046 (65.1) | 1,130 (57.0) | 16,828 (48.3) | <.001 |
| High risk results | 1,768 (28.5) | 545 (27.5) | 6,591 (18.9) | <.001 |
| Low risk results | 1,936 (31.2) | 486 (24.5) | 8,415 (24.2) | <.001 |
| **Functional imaging ischemia test, n (%)** |  |  |  |  |
| Test performed | 3,806 (61.3) | 1,070 (53.9) | 16,090 (46.2) | <.001 |
| High risk results | 1,798 (28.9) | 518 (26.1) | 7,096 (20.4) | <.001 |
| Low risk results | 1,874 (30.2) | 509 (25.7) | 8,300 (23.8) | <.001 |
| **Mean cholesterol value in the past 3 years, mmol/L ± SD*** |  |  |  |  |
| Total cholesterol | 4.28 ± 1.18 | 4.36 ± 1.19 | 4.39 ± 1.18 | <.001 |
| Non-HDL cholesterol | 3.14 ± 1.15 | 3.14 ± 1.15 | 3.18 ± 1.13 | 0.012 |
| LDL-cholesterol | 2.37 ± 0.98 | 2.39 ± 1.00 | 2.45 ± 0.99 | <.001 |
| HDL-cholesterol | 1.14 ± 0.31 | 1.22 ± 0.34 | 1.21 ± 0.36 | <.001 |
| Triglycerides | 1.76 ± 1.27 | 1.71 ± 1.14 | 1.69 ± 1.32 | 0.002 |
|  |  |  |  |  |
| **Mean blood glucose (HbA1c) in the past 3 years mmol/L ± SD** ** | 6.67 ± 1.37 | 6.44 ± 1.24 | 6.18 ± 1.17 | <.001 |
| **Mean fasting glucose in the past 3 years mmol/L ± SD ***** | 6.56 ± 2.21 | 6.32 ± 2.19 | 6.34 ± 2.10 | <.001 |
|  |  |  |  |  |
| **Medication use for patients > 65 years in the past 90 days prior to procedure, n (%) ****** |  |  |  |  |
| Angiotensin converting enzyme inhibitor | 618 (29.7) | 180 (24.0) | 5,798 (35.3) | <.001 |
| Angiotensin receptor blocker | 712 (34.2) | 269 (35.9) | 3,608 (22.0) | <.001 |
| Beta Blocker | 1,037 (49.8) | 339 (45.3) | 7,463 (45.4) | <.001 |
| Statin | 1,507 (72.4) | 492 (65.7) | 9,919 (60.4) | <.001 |
| Oral diabetic medication | 802 (38.5) | 224 (29.9) | 3,513 (21.4) | <.001 |
| Insulin | 177 (8.5) | 45 (6.0) | 960 (5.8) | <.001 |
| Abbreviation: SD, standard deviation |  |  |  |  |
| * Total cholesterol information available on 38,602 patients (89.7% of male cohort) | | | |  |
| ** HbA1C information available on 35,078 patients (81.5% of male cohort) | |  |  |  |
| *** Fasting glucose information available on 28,838 (67.0% of male cohort) | |  |  |  |
| **** Medication information based on 2,082 South Asians patients, 749 East Asian patients, and 16,427 White patients older than 65 years | | | |  |
